# A Systematic Review of the Predictive Value of Plasma D-Dimer Levels for Predicting Stroke Outcome

**DOI:** 10.3389/fneur.2021.693524

**Published:** 2021-07-06

**Authors:** Peng Zhang, Chun Wang, Junhua Wu, Shiliang Zhang

**Affiliations:** ^1^Department of Neurology, Zaozhuang Municipal Hospital, Zaozhuang, China; ^2^Department of Cardiology, Zaozhuang Hospital of Traditional Chinese Medicine, Zaozhuang, China; ^3^Department of Cardiovascular and Cerebrovascular, Zaozhuang Hospital of Traditional Chinese Medicine, Zaozhuang, China

**Keywords:** D-dimer, cerebrovascular accident, prognosis, morbidity, mortality

## Abstract

**Background:** Stroke is a leading cause of morbidity and mortality. Over the past decade, plasma D-dimer levels have emerged as a biomarker for predicting stroke outcome. However, no consensus in the literature currently exists concerning its utility for predicting post-stroke functional outcome and mortality.

**Objective:** To systematically review the effectiveness of plasma D-dimer levels for predicting functional outcome and mortality following stroke.

**Methods:** Five academic databases were screened according to PRISMA guidelines for eligible studies. With these studies, we conducted a random-effect meta-analysis to evaluate the impact of plasma D-dimer levels for predicting functional outcome and mortality post-stroke. We also conducted subgroup analyses to evaluate differences in predictive capacity for different stroke subtypes.

**Results:** Nineteen studies were included, containing data on 5,781 stroke patients (mean age: 65.26 ± 6.4 years). Overall methodological quality for the included studies was high. Meta-analysis showed that increased D-dimer levels were predictive of worsened functional outcomes (Hazard ratio: 2.19, 95% CI: 1.63–2.93) and elevated overall mortality (2.29, 1.35–3.88). Subgroup analysis showed that plasma D-dimer levels were more predictive of poorer functional outcomes for ischemic (2.08, 1.36–3.18) stroke as compared to intracerebral hemorrhage (2.62, 1.65–4.17). We also noted that predictive capacity was similar when it came to mortality in patients with cryptogenic ischemic stroke (2.65, 0.87–8.08) and intracerebral hemorrhage (2.63, 1.50–4.59).

**Conclusion:** The study provides preliminary evidence concerning the capacity of plasma D-dimer levels for predicting functional outcomes and mortality following stroke and reports that higher D-dimer levels of are associated with poorer functional outcomes and higher mortality.

## Introduction

Stroke is the second most common cause of death or disability worldwide (1, 2). Characterized as a cerebrovascular accident that hampers blood flow resulting in brain damage (3), stroke accounts for almost 5.5 million deaths and 116.4 million disability-adjusted life-years per year (4, 5).

Brain structural damage in stroke patients occurs due to either blood vessel occlusion or intracerebral hemorrhage (6, 7). The resultant ischemic damage then initiates a signaling cascade that triggers excitotoxic and/or inflammatory mechanisms eventually resulting in cellular apoptosis (8). Studies suggest that hemodynamic restoration is the primary mode for limiting neural injury (9, 10). However, this approach does not completely eliminate morbidity and mortality (7, 11). As such, preemptive diagnosis is imperative and is widely recommended (12–16).

D-dimers, such as circulating fibrin-degradation products, have recently been shown to be critical for predicting short- and long-term stroke-related outcomes (12, 17, 18). The presence of D-dimers can be representative of total fibrin concentrations, thereby serving as a biomarker for intravascular fibrinolysis and intravascular thrombus formation (19, 20). For stroke patients, this biomarker can detect disrupted vessels, dissolved clots, and the release of stroke-related tissue factors. D-dimers also serve as a good biomarker because of its prolonged stability, half-life, cost-effectiveness, and high sensitivity (> 97%) (21–24).

To date, only a few individual retrospective cohort studies have attempted to evaluate whether plasma D-dimer levels can predict future functional outcomes and mortality post-stroke (25–28). These studies have not established a consensus here. While some studies reported a positive correlation between mortality and plasma D-dimer levels (29–32), others have reported weaker or no correlation (27, 33, 34). Similarly, there is also no consensus concerning whether D-dimer levels are predictive for overall functional outcome. Some studies noted that plasma D-dimer levels were related to worse functional outcomes (26, 31, 33, 35), other have reported limited correlations (25, 28). To date, we have located one systematic review that attempted to evaluate the predictive capacity for plasma D-dimers (12). However, this review failed to include a meta-analysis. Moreover, since it was published in 2009, an update centered around the current evidence is strongly warranted. While a recently published meta-analysis did attempt to evaluate the prognostic impact of plasma D-dimer levels on mortality, it only contained two studies (17). We therefore, in this present systematic review and meta-analysis, attempt to evaluate the capacity for plasma D-dimer levels to predict post-stroke functional outcome and mortality.

## Methods

### Data Search Strategy

The database search for this meta-analysis was done according to PRISMA (Preferred Reporting Items for Systematic Reviews and Meta-Analyses) guidelines (36). Five databases (Web of Science, MEDLINE, CENTRAL, EMBASE, and Scopus) were screened for studies published prior to February 2021. The search was performed across a combination of MeSH keywords, including “D-dimer,” “stroke,” “intracerebral stroke,” “ischemic stroke,” “cryptogenic stroke,” “subarachnoid stroke,” “hemorrhage,” “cerebrovascular disease,” “cerebrovascular accident,” “functional outcome,” and “mortality.” A sample search strategy for EMBASE database has been provided in Supplementary Table 1. References cited in included studies were manually examined to identify further relevant hits. Study inclusion criteria were as follows:

- Studies evaluating the impact of D-dimer levels in population groups following stroke.
- Studies evaluating functional outcome and mortality outcome.
- Studies investigating human participants.
- Case-control studies, prospective trials, or retrospective cohort trials.
- Studies published in peer-reviewed scientific journals.
- Studies published in English.

Study screening and data collection was independently conducted by two reviewers. The extraction of data was done manually while using Microsoft excel. In cases of disagreements concerning eligibility of studies, discussions were held with a third independent reviewer. Moreover, in conditions where required data was not mentioned in the included studies, repeated attempts were made to contact respective corresponding authors for additional data. We extracted the following data from the included studies: author information, country of research, type of study, descriptive data of the sample, type of cerebrovascular incident, baseline D-dimer levels, functional outcomes, and mortality outcomes.

### Quality Assessment

Risk of bias appraisal for included studies was performed using Cochrane's risk of bias assessment tool for non-randomized controlled trials (37). This tool evaluates study outcomes for possible selective reporting, confounding bias, measurement of outcomes, and incomplete data availability. Appraisal was carried out by two reviewers, with a third reviewer called in to arbitrate in case of disagreement. In addition, we also assessed the overall level of evidence presented in the literature by using Oxford Centre for Evidence Based Medicine tool (38).

### Data Analysis

This study performed a within-group meta-analysis using Comprehensive Meta-analysis (CMA) software version 2.0 (39). This meta-analysis was conducted based on a random-effects model (40). Hazard ratios were calculated to determine the impact of D-dimer levels on functional outcomes and mortality following stroke. Heterogeneity among studies was assessed using *I*^2^ statistics (0–25%: negligible heterogeneity, 25–75%: moderate heterogeneity, and ≥75%: substantial heterogeneity) (41). To ensure clinical heterogeneity we also carried out subgroup analyses on the basis of stroke subtypes i.e., intracerebral hemorrhage, subarachnoid hemorrhage, central nervous system infarction (including ischemic stroke and silent infarction). Besides, we also carried out subgroup analyses for two studies reporting the outcomes of cryptogenic ischemic stroke (i.e., a subtype of ischemic stroke). In the included studies cryptogenic ischemic stroke was defined as per the TOAST criteria which defines it as a brain infarction that is not attributable to a definite cardioembolism, large artery atherosclerosis, or small artery disease despite extensive vascular, cardiac, and serologic evaluation (42). Publication bias was evaluated using Duval and Tweedy's trim and fill procedure (43), which examines publication bias by adding studies on either side of the plotted graph. The significance level for this study was determined at 5%.

## Results

**Figure 1:**
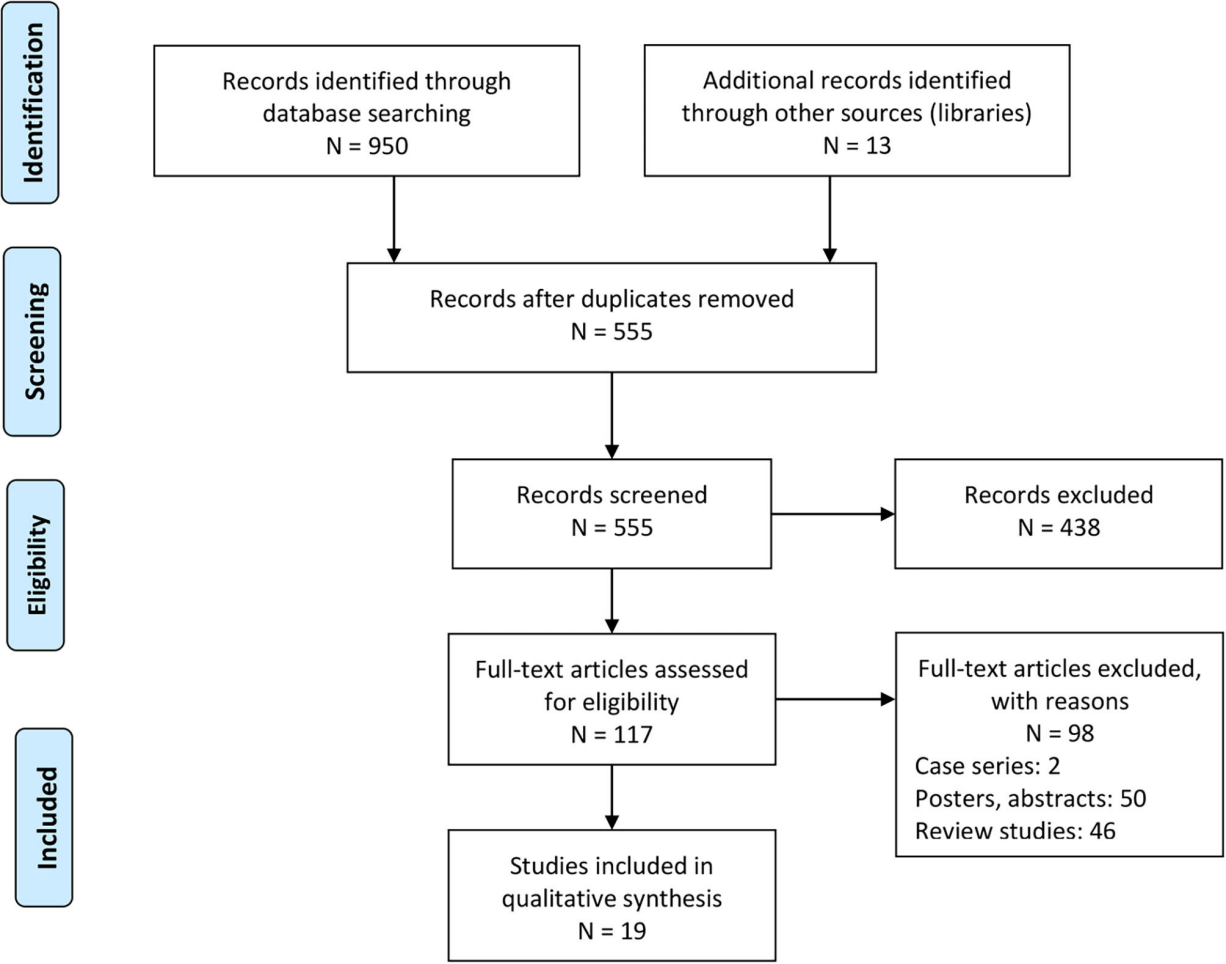
PRISMA flowchart for study inclusion.

Database screening yielded 950 studies, while manual screening added another 13 to this total. After applying inclusion criteria, 19 studies remained (Figure 1). Thirteen of these were retrospective cohort studies (25–28, 30–35, 44, 45), while the other six were prospective cohort studies (29, 46–50). Relevant data from each study was extracted and tabulated (Table 1).

**Table 1:** Study details.

**References**	**Country**	**Type of study**	**Sample descriptive**	**Age (M ± S.D years)**	**Type of cerebrovascular stroke**	**D-dimer recorded**	**Assessment method of functional outcome**	**Follow-up functional outcome**	**D-dimer levels**	**Functional outcome (Hazard ratio, 95% CI, *p*-value)**	**Follow-up mortality**	**Mortality (Hazard ratio, 95% CI, *p*-value)**
Hou et al. (46)	China	Prospective cohort study	10,518 (3,283F, 7,235M)	62.3 ± 11.4	Ischemic	At admission	Modified Rankin scale score ≥ 3	12 months	1.1 μg/mL	1.59 (1.32–1.91, <0.001)	–	–
Ye et al. (50)	China	Prospective cohort study	236 (91F, 145M)	70	Ischemic	At admission	Modified Rankin scale score > 2	1-month	0.45 mg/L	2.07 (1.49–2.88, <0.001)	–	–
Liu et al. (47)	China	Prospective cohort study	489	70.1 ± 11.9	Ischemic	–	–	6 months	1.83 ± 2.29 mg/L	–	–	3.06 (1.61–5.83, <0.001)
Sato et al. (48)	Japan	Prospective cohort study	130	–	Ischemic	At admission	Modified Rankin scale score ≥ 3	3 months	–	3.31 (1.14–9.61, <0.028)	–	–
Wang et al. (49)	China	Prospective cohort study	1,485 (997F, 488M)	63.9 ± 12.7	Ischemic	At admission	Modified Rankin scale score ≥ 3	3 months	0.93 ± 45.8 mg/L	2.93 (1.91–4.50, <0.0001)	–	–
Zhou et al. (28)	China	Retrospective cohort study	1,332 (694F, 638M)	65 ± 14	Intracerebral	1-h post admission	Modified Rankin scale score ≥ 3	3 months	–	1.48 (1.08–2.06, 0.1)	3 months	2.01 (1.18–3.42, 0.1)
Hutanu et al. (35)	Romania	Retrospective cohort study	89	71.9 ± 10	–	At admission	Modified Rankin scale score ≥ 3	3 months	185.1 (185.06–245.06) ng/mL	8.3 (1.4–47.6, 0.01)	–	–
Nezu et al. (27)	Japan	Retrospective cohort study	295 (143F, 152M)	72 ± 13	Cryptogenic ischemic stroke	–	–	–	–	–	36 months	1.35 (0.74–2.5, 0.33)
Fukuda et al. (25)	Canada	Retrospective cohort study	187 (37F, 150M)	62.45	Aneurysm, subarachnoid hemorrhage, intracerebral, intraventricular	At admission	Modified Rankin scale score ≥ 3	3 months	–	1.5 (1.1–2.0, 0.003)	–	–
Liu et al. (26)	China	Retrospective cohort study	146 (89F, 57M)	57	Subarachnoid hemorrhage	At admission	Glasgow coma scale, world Federation of Neurosurgical Societies stage IV to V	6 months	–	2.67 (1.66–4.45, <0.01)	–	–
Hsu et al. (44)	Taiwan	Retrospective cohort study	347 (140F, 207M)	67.6 ± 13.1	Intracerebral	24-h post stroke	Modified Rankin scale score ≥ 3	3 months	–	1.9 (1.27–2.86, 0.002)	–	–
Chen et al. (29)	Taiwan	Prospective cohort study	43 (14F, 29M)	56.6 ± 15	Intraventricular	At admission	–	–	43.1 ± 45.8 μg/mL	–	–	30 (3–295, 0.0006)
Kim et al. (32)	South Korea	Retrospective cohort study	570 (214F, 356M)	60.8 ± 13.6	Cryptogenic ischemic stroke	At admission	–	–	–	–	34.0 ± 22.8 months	4.28 (1.79 – 10.27, 0.001)
Hu et al. (33)	China	Retrospective cohort study	259 (98F, 161M)	58 ± 14	Subarachnoid hemorrhage, intracerebral, intraventricular	At admission	Modified Rankin scale score ≥ 3	3 months	–	2.72 (1.13–6.59, 0.02)	7 days	1.23 (1.01–1.50, 0.033)
Yang et al. (51)	China	Prospective cohort study	220 (93F, 127M)	68	Ischemic	At admission	Modified Rankin scale score ≥ 3	3 months	1.36 (0.55–3.11) mg/L	4.25 (1.93–9.28, 0.001)	–	–
Chiu et al. (30)	Taiwan	Retrospective cohort study	170	65.9 ± 12.6	Intracerebral	At admission	Glasgow coma scale ≥ 2	72 h	1,231.9 ± 1,595.5 ng/mL	–	30 days	2.72 (1.08–6.9, 0.002)
Krarup et al. (45)	Norway	Retrospective cohort study	449 (218F, 231M)	80	Ischemic	–	Scandinavian stroke scale ≥ 3	48 h	–	0.99 (0.97–1.01, 0.34)	–	–
Üstündag et al. (34)	Turkey	Retrospective cohort study	91 (49F, 42M)	64.5 ± 12.7	–	–	–	–	–	–	–	0.51 (0.32–0.79, 0.003)
Delgado et al. (31)	Spain	Retrospective cohort study	98 (35F, 63M)	61–80	Intracerebral	At admission	NIH Stroke Scale ≥ 4	48 h	1,780 (354–2,655) ng/mL	6.8 (1.2–36.9, 0.02)	3 months	8.7 (1.4–54.1, 0.02)

### Participant Information

The 19 included studies featured data from 5,781 total patients (2,821 females and 2,701 males). Four studies did not report gender distributions (30, 35, 47, 48). Average patient age was 65.26 ± 6.4 years, with one study reporting age as only a range (31) and one omitting age altogether (48).

### Quality Assessment for Included Non-randomized Controlled Trials

**Figure 2:**
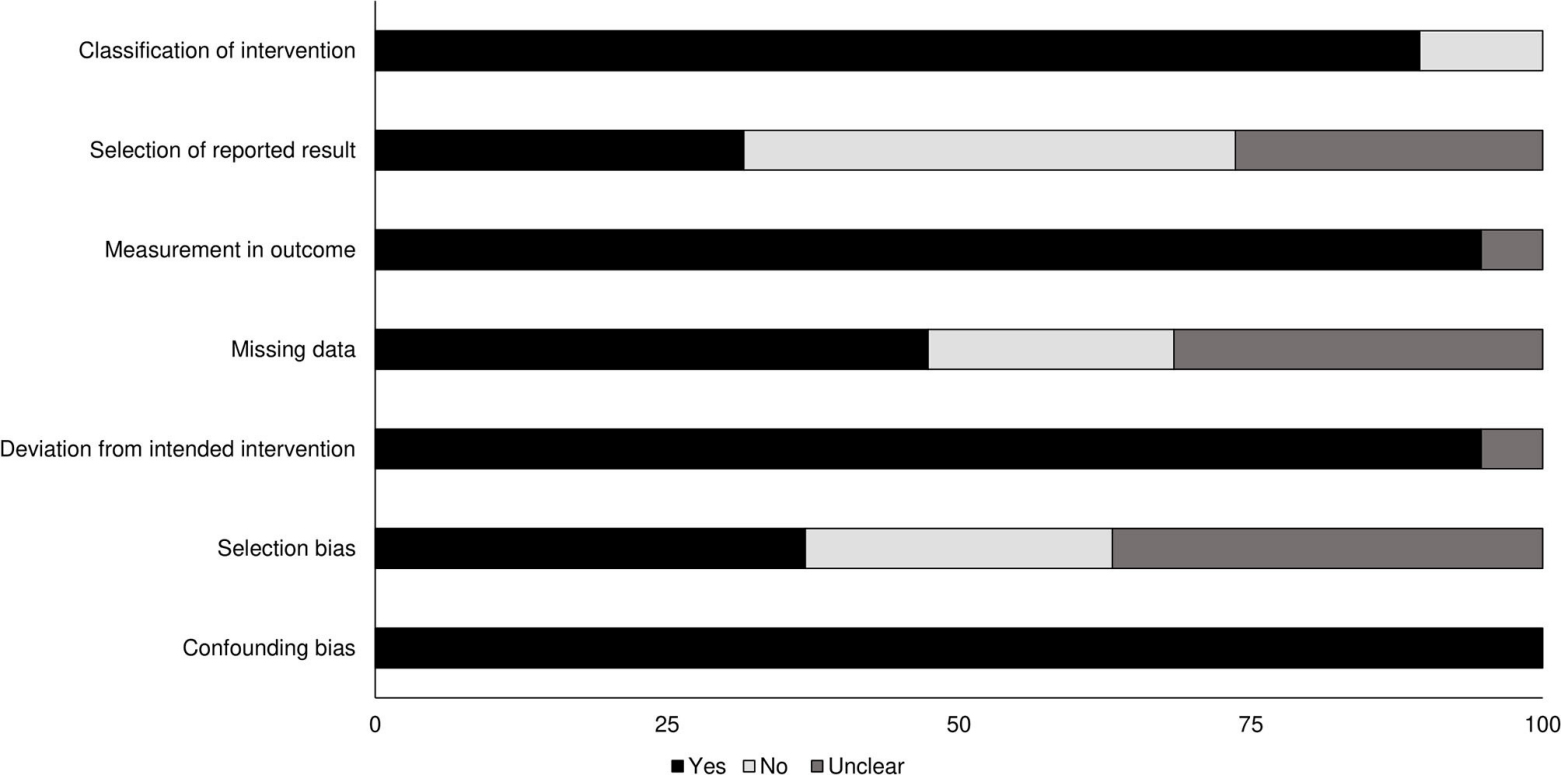
Risk of bias for non-randomized controlled trials according to the Cochrane risk of bias assessment.

Risk of methodological bias for the included non-randomized controlled trials was assessed with the ROBINS-I tool (Table 2). Overall risk among the included studies was low, with missing data, selection of reported results, and selection bias the most prominent aspects (Figure 2). We also found that the overall level of evidence according to the Oxford Centre for Evidence Based Medicine to be 2b.

**Table 2:** Risk of bias according to Cochrane's risk of bias assessment tool for included non-randomized controlled trials.

**References**	**Confounding bias**	**Selection bias**	**Deviation from intended intervention**	**Missing data**	**Measurement in outcome**	**Selection of reported result**	**Classification of intervention**	**Level of evidence**
Hou et al. (46)	+	+	+	?	+	–	+	2b
Ye et al. (50)	+	+	+	?	+	–	+	2b
Liu et al. (47)	+	+	+	?	+	–	+	2b
Sato et al. (48)	+	–	+	?	+	–	+	2b
Wang et al. (49)	+	+	?	+	+	?	+	2b
Zhou et al. (28)	+	+	+	+	+	+	+	2b
Hutanu et al. (35)	+	–	+	+	?	–	+	2b
Nezu et al. (27)	+	?	+	–	+	?	+	2b
Fukuda et al. (25)	+	?	+	–	+	?	+	2b
Liu et al. (26)	+	–	+	?	+	–	+	2b
Hsu et al. (44)	+	?	+	?	+	+	+	2b
Chen et al. (29)	+	?	+	–	+	?	+	2b
Kim et al. (32)	+	?	+	+	+	+	+	2b
Hu et al. (33)	+	+	+	+	+	+	–	2b
Yang et al. (51)	+	+	+	+	+	+	+	2b
Chiu et al. (30)	+	?	+	+	+	+	+	2b
Krarup et al. (45)	+	?	+	–	+	?	+	2b
Üstündag et al. (34)	+	–	+	+	+	–	–	2b
Delgado et al. (31)	+	–	+	+	+	–	+	2b

### Publication Bias

**Figure 3:**
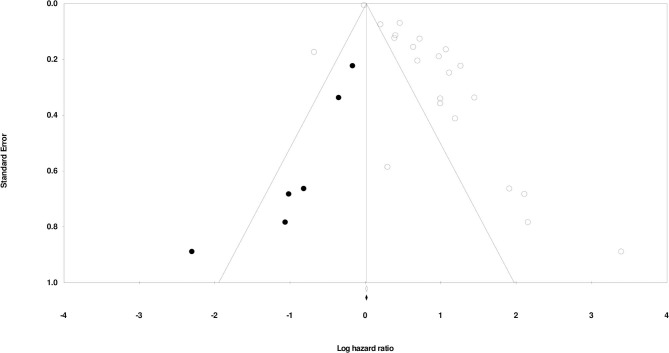
Publication bias as determined using Duval & Tweedy's trim and fill method.

Duval and Tweedy's trim and fill method was used to determine if studies were missing from either side of the mean effect. The method observed that six studies were missing on the left side of the mean effect. The overall random effects model determined point estimates and 95% confidence intervals for all studies combined as 2.13 (95% CI: 1.69–2.67). Imputed point estimate using the trim and fill method was 1.74 (95% CI: 1.41–2.15) (Figure 3).

### Meta-analysis Report

#### Functional Outcomes

**Figure 4:**
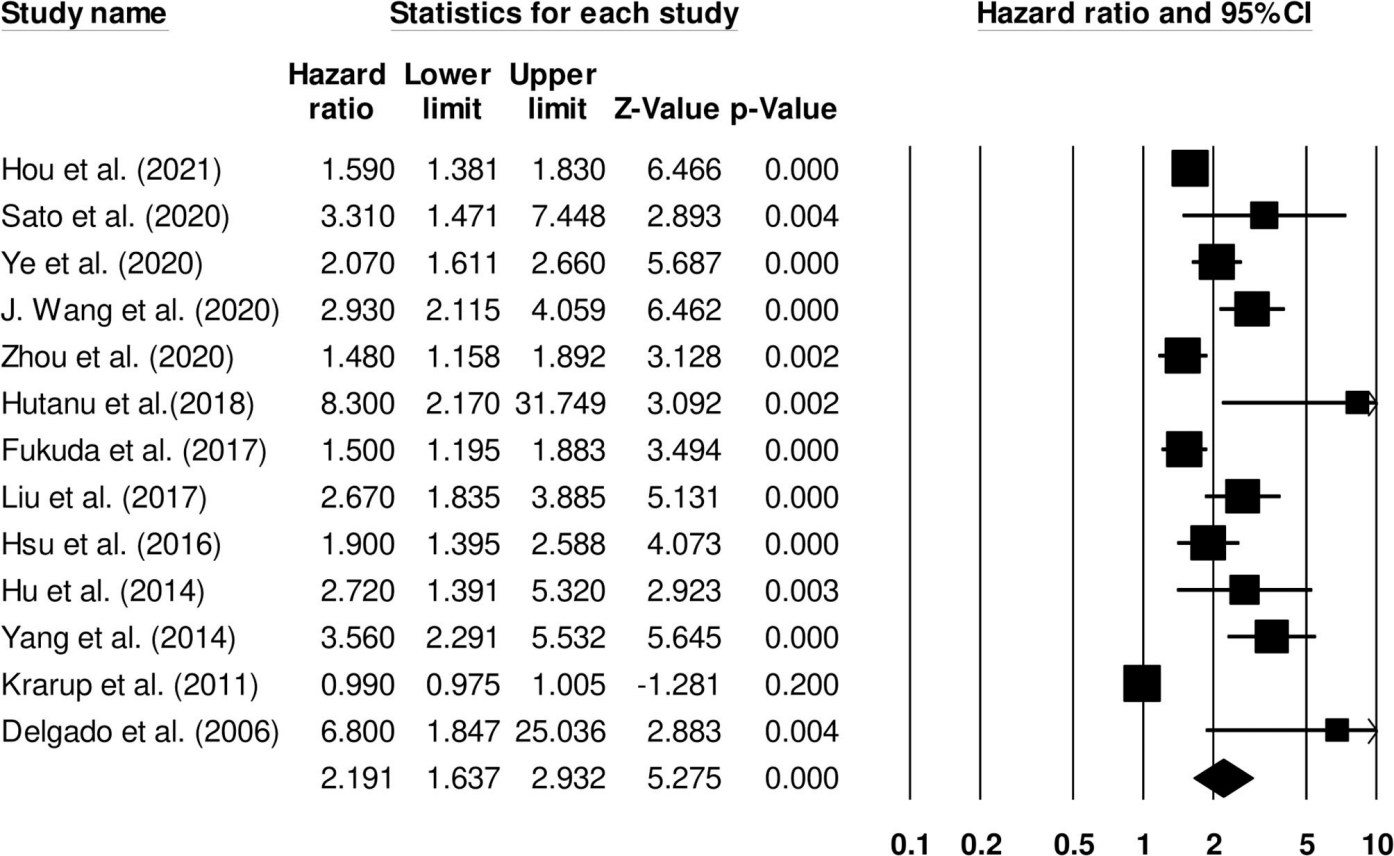
Forest plot for studies evaluating the impact of D-dimer level on post-stroke functional outcomes. Hazard ratios are presented as black boxes while 95% confidence intervals are presented as whiskers. A small hazard ratio represents a lower influence of D-dimer levels on stroke patient functional outcome while a higher hazard ratio represents a higher influence.

Thirteen studies examined the impact of D-dimer levels on post-stroke functional outcome (25, 26, 28, 31, 33, 35, 44, 49, 51). Hazard ratio was 2.19 (95% CI: 1.63–2.93, *p* < 0.001) with no heterogeneity (*I*^2^: 0%) (Figure 4).

**Figure 5:**
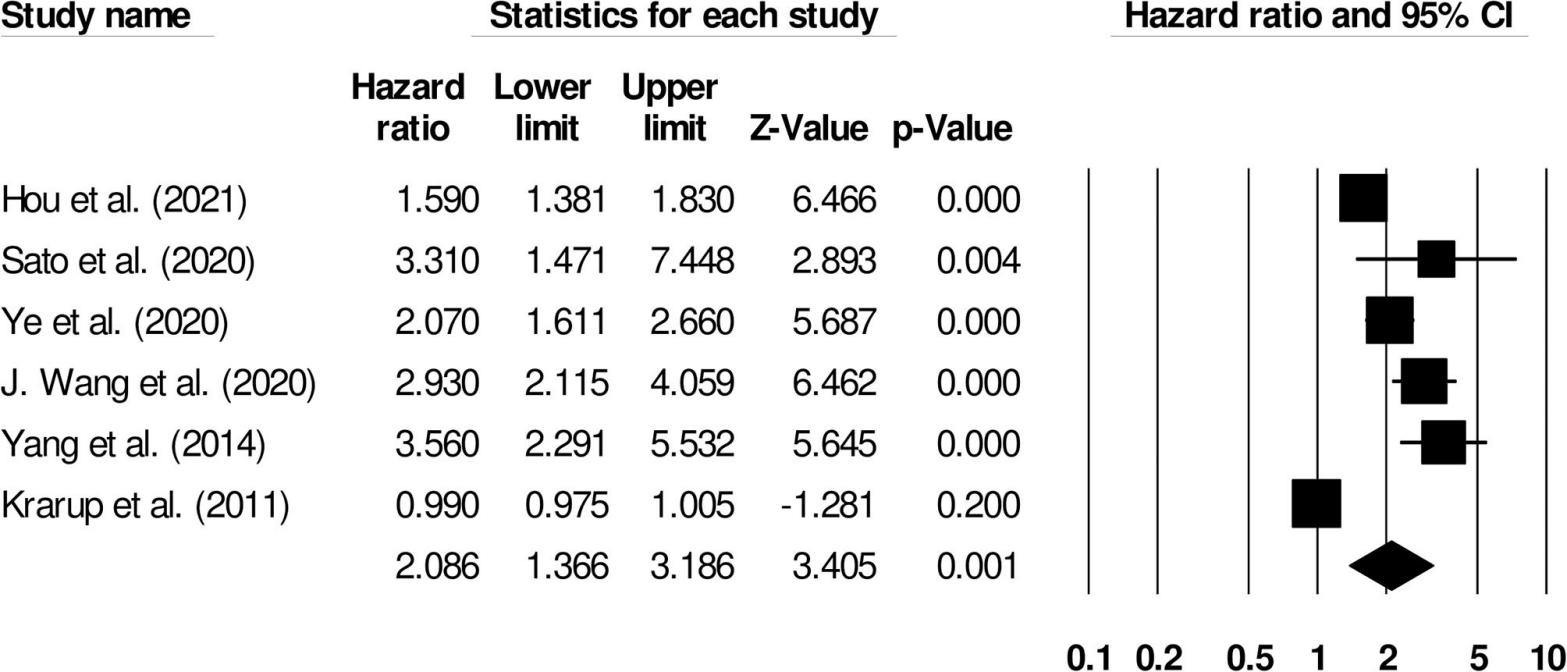
Forest plot for studies evaluating the impact of D-dimer level on post-ischemic stroke functional outcomes. Hazard ratios are presented as black boxes while 95% confidence intervals are presented as whiskers. A small hazard ratio represents a lower influence of D-dimer levels on stroke patient functional outcome while a higher hazard ratio represents a higher influence.

**Figure 6:**
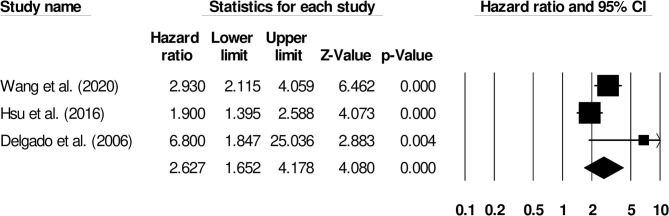
Forest plot for studies evaluating the impact of D-dimer level on post-intracerebral hemorrhage functional outcomes. Hazard ratios are presented as black boxes while 95% confidence intervals are presented as whiskers. A small hazard ratio represents a lower influence of D-dimer levels on stroke patient functional outcome while a higher hazard ratio represents a higher influence.

Further subgroup analysis for functional outcome post-stroke was carried out to examine the effect of stroke type. Six studies reported functional outcomes for patients with ischemic stroke (Hazard ratio: 2.08, 95% CI: 1.36–3.18, *p* = 0.001; *I*^2^: 0%; Figure 5) while three included studies evaluated outcomes for intracerebral hemorrhage patients with negligible heterogeneity (Hazard ratio: 2.62, 95% CI: 1.65–4.17, *p* = 0.001; *I*^2^: 23.52%; Figure 6).

**Figure 7:**
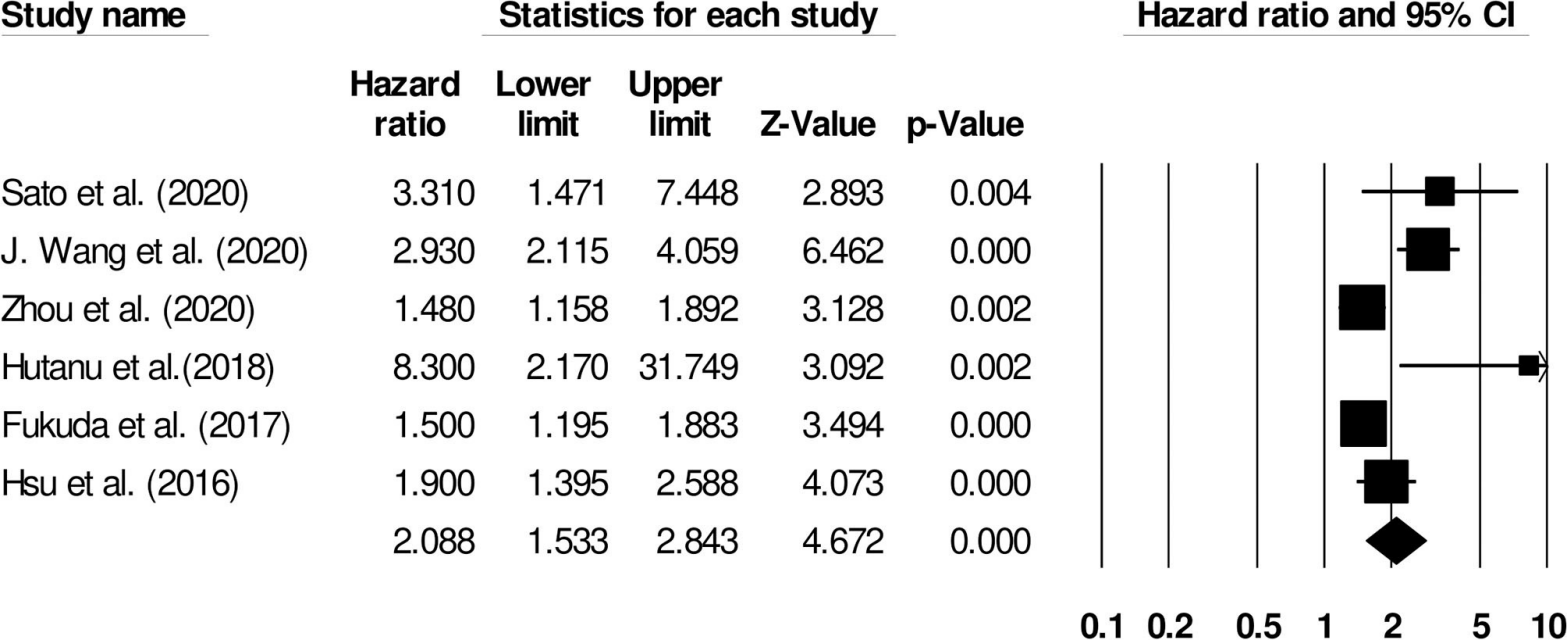
Forest plot for studies evaluating the impact of D-dimer level on post-stroke functional outcomes for 3 months follow up. Hazard ratios are presented as black boxes while 95% confidence intervals are presented as whiskers. A small hazard ratio represents a lower influence of D-dimer levels on stroke patient functional outcome while a higher hazard ratio represents a higher influence.

**Figure 8:**
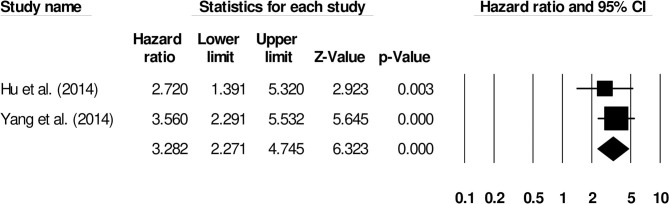
Forest plot for studies evaluating the impact of D-dimer level on post-intracerebral hemorrhage functional outcomes for 2 months follow up. Hazard ratios are presented as black boxes while 95% confidence intervals are presented as whiskers. A small hazard ratio represents a lower influence of D-dimer levels on stroke patient functional outcome while a higher hazard ratio represents a higher influence.

We also conducted two subgroup analyses based on different follow-up periods and assessment methods. Firstly, we identified only six studies that had reported a uniform follow-up of 3 months and they had used modified rankin scale for assessing functional outcome. We observed increased mortality outcomes for patients with moderate heterogeneity (Hazard ratio: 2.08, 95% CI: 1.53–2.84, *p* < 0.001; Figure 7; *I*^2^:31.1%). Secondly, we identified two studies that had reported a uniform follow-up of 2 months and they had also used modified rankin scale for assessing functional outcome. We observed increased mortality outcomes for patients with no heterogeneity (Hazard ratio: 3.28, 95% CI: 2.27–4.74, *p* < 0.001; Figure 8; *I*^2^:0%).

#### Mortality Outcomes

**Figure 9:**
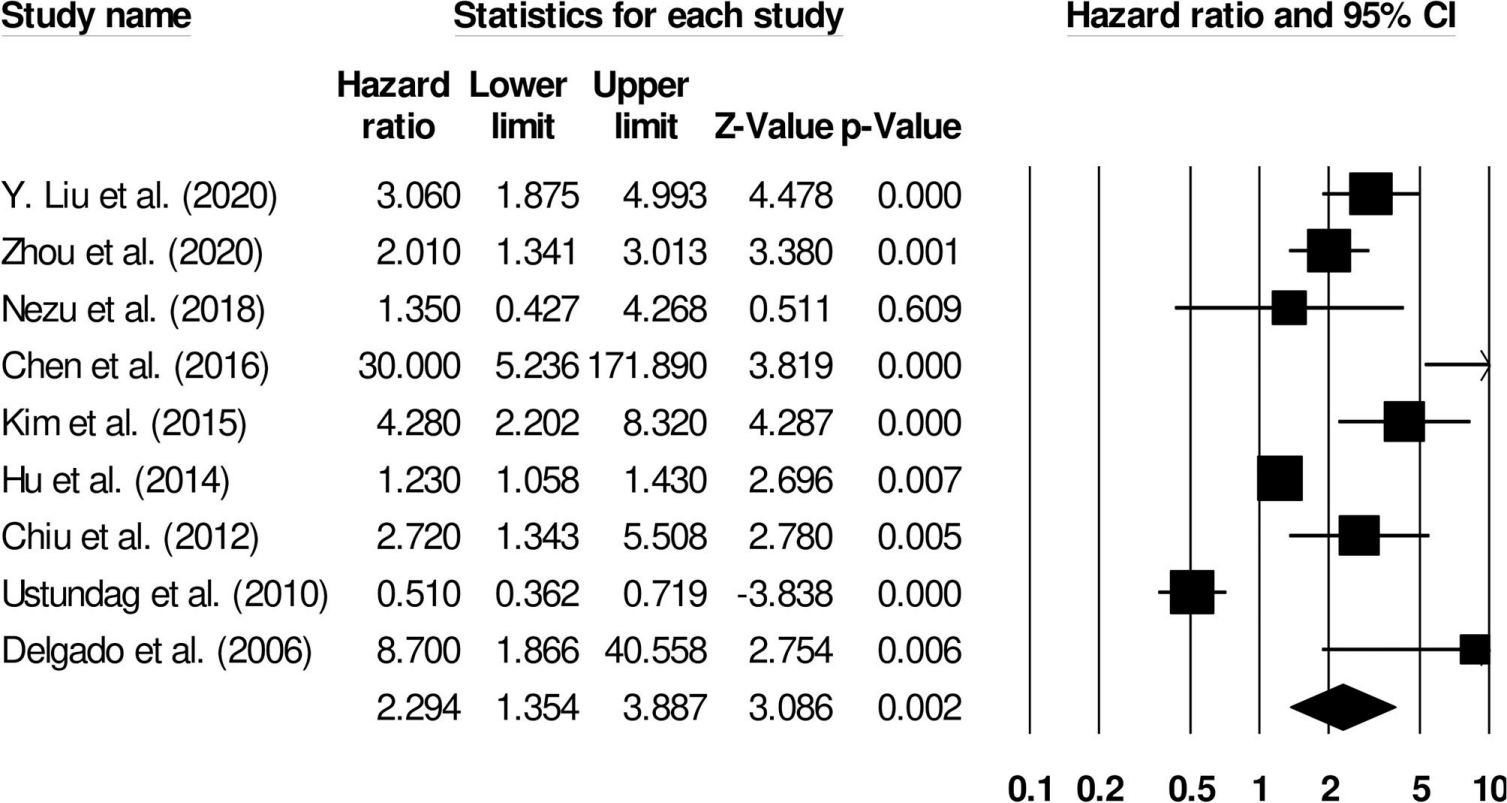
Forest plot for studies evaluating the impact of D-dimer level on post-stroke mortality outcomes. Hazard ratios are presented as black boxes while 95% confidence intervals are presented as whiskers. A small hazard ratio represents a lower influence of D-dimer levels on stroke patient functional outcome while a higher hazard ratio represents a higher influence.

Nine studies evaluated the impact of D-dimer levels on post-stroke mortality (26–34). A hazard ratio of 2.29 (95% CI: 1.35–3.88, *p* = 0.002, Figure 9) was observed, with moderate heterogeneity (*I*^2^: 39.03%).

**Figure 10:**
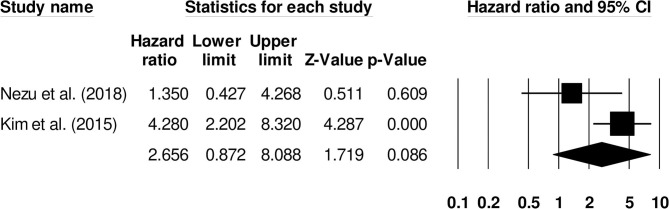
Forest plot for studies evaluating the impact of D-dimer level on post-cryptogenic ischemic stroke mortality outcomes. Hazard ratios are presented as black boxes while 95% confidence intervals are presented as whiskers. A small hazard ratio represents a lower influence of D-dimer levels on stroke patient functional outcome while a higher hazard ratio represents a higher influence.

**Figure 11:**
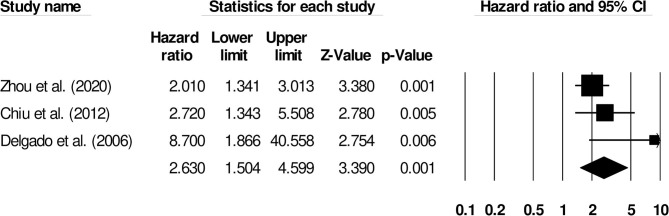
Forest plot for studies evaluating the impact of D-dimer level on post-intracerebral hemorrhage mortality outcomes. Hazard ratios are presented as black boxes while 95% confidence intervals are presented as whiskers. A small hazard ratio represents a lower influence of D-dimer levels on stroke patient functional outcome while a higher hazard ratio represents a higher influence.

Further subgroup analysis for overall mortality was carried out examining the impact of stroke type. Two studies reported mortality outcomes for patients with cryptogenic ischemic stroke (Hazard ratio: 2.65, 95% CI: 0.87–8.08, *p* = 0.08; Figure 10; *I*^2^: 0%) while three included studies evaluated mortality outcomes for intracerebral hemorrhage patients with negligible heterogeneity (Hazard ratio: 2.63, 95% CI: 1.50–4.59, *p* = 0.001; Figure 11; *I*^2^: 18.8%).

**Figure 12:**
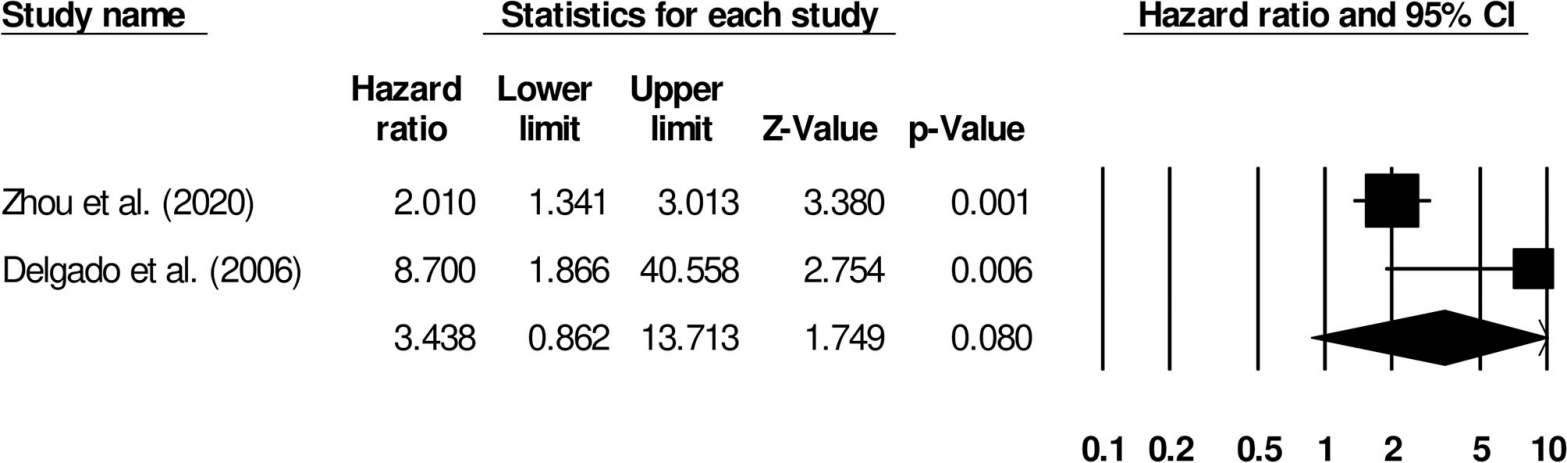
Forest plot for studies evaluating the impact of D-dimer level on post-stroke mortality outcomes at 3 months follow up. Hazard ratios are presented as black boxes while 95% confidence intervals are presented as whiskers. A small hazard ratio represents a lower influence of D-dimer levels on stroke patient functional outcome while a higher hazard ratio represents a higher influence.

We also conducted subgroup analyses based on different follow-up periods. Here, we identified only two studies that had reported a uniform follow-up of 3 months. We observed increased mortality outcomes for patients (Hazard ratio: 3.43, 95% CI: 0.86–13.71, *p* = 0.08; Figure 12; *I*^2^: 0%).

## Discussion

This systematic review and meta-analysis suggest that poorer functional outcome and increased mortality incidence following stroke is associated with increased plasma D-dimer levels. We also noted that the association between plasma D-dimer levels and functional outcomes was stronger for ischemic stroke than intracerebral hemorrhage. However, plasma D-dimer predictive capacity for mortality between patients with cryptogenic ischemic stroke and intracerebral hemorrhage was similar.

Stroke management is challenging because of its atypical pathophysiology, poor prognosis, and heterogeneous manifestation (52, 53). In this light, preemptive prediction through biomarker detection has been widely recommended (54–56). Plasma D-dimer levels has been identified as a biomarker that was sensitive and specific for predicting short- and long-term functional outcomes, recurrence, and mortality post-stroke (12, 57). Johnson et al. (19) reported that D-dimer levels are indirectly indicative of hemostasis and thrombosis incidence. Furthermore, plasma D-dimers levels can be used to categorize increased risk for thromboembolic disorders (57, 58). Elevated plasma D-dimers could potentially boost interleukin-1 and 6 production (17, 59) precipitating worsened prognostic outcome following stroke (60). Nonetheless, despite pertaining several positive aspects, the routine use of plasma D-dimer in the current medical setting is complicated by its non-specificity. For instance, the plasma D-dimer levels are also susceptible to different inflammatory states, presence of infection, cancer, and venous thromboembolism (58, 61, 62). Therefore, the presence of a high plasma D-dimer at times could serve as a false positive with respect to stroke. Moreover, the clinical utility of plasma D-dimer is also limited perhaps because of limited clinical awareness this biomarker has in a stroke setting (i.e., plasma D-dimer evaluation not routinely demanded) (63).

This systematic review observed that plasma D-dimer levels could predict post-stroke functional outcome. These findings are aligned with other studies. Zhou et al. (28) showed that elevated plasma D-dimer levels measured 1-h post-hospital admission could predict poor 3-month functional outcomes for stroke patients with high precision and developed a scoring system for clinical practice. Furthermore, Hutanu et al. (35) found that plasma D-dimers could independently predict poor functional outcome in ischemic stroke patient outcomes whereas plasma c-reactive protein, neutrophil gelatinase associated lipocalin, the soluble receptor of tumor necrosis factor alfa, and neuron specific enolase could not.

We also examined the ability of plasma D-dimer levels to predict post-stroke mortality. The majority of included studies noted that plasma D-dimer levels were predictive for mortality. Hu et al. (33), for instance, noted that plasma D-dimer levels reliably predicted 7-day mortality with almost 88% sensitivity and 68% specificity—albeit the authors did note that plasma D-dimers were not as efficient as the standard Glasgow Coma scale. Similarly, Nezu et al. (27) reported that plasma D-dimer levels recorded at admission not only correlated with the National Institute of Health Stroke Scale but also with mortality. It is possible that high plasma D-dimer levels may be predictive of post-stroke mortality because it can also capture conditions such as venous thrombus, malignancy, or atrial fibrillation (64). In a novel study, Chen et al. (29) found that cerebrospinal fluid D-dimer levels were highly sensitive (88%) and specific (81%) for predicting 30-day mortality in stroke patients. The authors suggest that cerebrospinal D-dimer levels could be used reliably in patients with intracerebral or intraventricular hemorrhage. Besides, in the subgroup analyses of mortality, we observed that the risks of mortality were higher for patients with cryptogenic ischemic stroke (i.e., 2.65) when compared with the overall analyses (i.e., 2.19). In our opinion, this difference could perhaps be attributed to the small number of studies included in the subgroup analysis of cryptogenic ischemic stroke (i.e., two studies).

This study is hampered by a few limitations. This study is not pre-registered in a systematic review repository such as PROSPERO York or the Joanna Briggs Institute (65). This was because the current COVID-19 pandemic crisis has extended registration queues to over 1 year. Besides, this review does not provide a list of studies that were excluded with reasoning. This was a major flaw on our behalf, and we request future studies to address this limitation. Additionally, because of data paucity, we were unable to carry out sub-group analyses for two important parameters: the relationship between functional outcome and stroke type and the relationship between plasma D-dimer levels and short- and long-term functional outcomes. Similarly, there was a huge discrepancy in the sample sizes between the studies we included (i.e., 10,518 participants in Hou et al., and 43 participants in Chen et al.). Additionally, although we conducted subgroup analyses based on the specific follow-up periods and assessment methodologies (i.e., for functional outcomes), we were only able to include studies that reported follow-up at 3 and 2 months. Other studies for instance had reported a varied range of follow-up (i.e., at 12 months, 1 month, 48 h, 72 h) and because these were only singular studies, we could not conduct subgroup analyses for them. We presume that this could be an important source of heterogeneity in the analyses we conducted and could possibly incur bias in our results. We therefore recommend future studies to focus on these areas where there is a knowledge gap.

## Conclusion

In conclusion, we provide preliminary 2b level of evidence concerning the capacity of plasma D-dimer levels for predicting stroke patient functional outcome and mortality. We show that increased plasma D-dimer levels are predictive of poorer functional outcomes and increased mortality. The findings from the present study may have wider implications in developing best practice guidelines for predicting post-stroke prognostic outcomes.

## Data Availability Statement

The original contributions presented in the study are included in the article/Supplementary Material, further inquiries can be directed to the corresponding author/s.

## Author Contributions

PZ designed the project. CW and JW were involved in data collection and data analysis. SZ prepared the manuscript. JW edited the manuscript. All authors contributed to the article and approved the submitted version.

## Conflict of Interest

The authors declare that the research was conducted in the absence of any commercial or financial relationships that could be construed as a potential conflict of interest.
