# Supplementary material for: A Systematic Review of the Predictive Value of Plasma D-Dimer Levels for Predicting Stroke Outcome
Source: Front Neurol. 2021 Jul 6;12:693524. doi: 10.3389/fneur.2021.693524 (PMC8289899; doi:10.3389/fneur.2021.693524)
Supplement: Supplementary file 1 [file Table_1.DOCX]

Supplementary table 1. Sample search strategy

| Database | EMBASE |
| --- | --- |
| Date | 02/02/2021 |
| Strategy | #1 AND #2 AND #3 AND #4 |
| #1 | (‘Stroke’ OR ‘Apoplexy’ OR ‘CVA’ OR ‘Cerebrovascular accident’ OR ‘Cerebral stroke’ OR ‘Cerebrovascular accident, acute’ OR ‘Stroke, acute’ OR ‘Vascular accident, brain’ OR ‘Cerebrovascular apoplexy’ OR ‘Cerebrovascular stroke’ OR ‘Cerebral infarction’ OR ‘Cerebral hemorrhage’ OR ‘Brain ischemia’ OR ‘Cryptogenic stroke’)/de OR (Stroke OR Apoplexy OR CVA OR Cerebrovascular accident OR Cerebral stroke OR Cerebrovascular accident, acute OR Stroke, acute OR Vascular accident, brain OR Cerebrovascular apoplexy OR Cerebrovascular stroke OR Cerebral infarction OR Cerebral hemorrhage OR Brain ischemia OR Cryptogenic stroke); ti,ab |
| #2 | (‘D-dimer’ OR ‘Plasma d-dimer’ OR ‘fibrin degradation product’ OR ‘FDP’)/de OR (D-dimer OR Plasma d-dimer OR fibrin degradation product OR FDP); ti,ab |
| #3 | (‘Case control study’ OR ‘Prospective cohort trial’ OR ‘Retrospective cohort trial’)/de OR (Case control study OR Prospective cohort trial OR Retrospective cohort trial); ti,ab |
| #4 | (‘Morbidity’ OR ‘Mortality’ OR ‘Age specific death rate’ OR ‘Crude death rate’ OR ‘Death rate’ OR ‘Excess mortality’ OR ‘Mortality rate’)/de OR (Morbidity OR Mortality OR Age specific death rate OR Crude death rate OR Death rate OR Excess mortality OR Mortality rate); ti, ab |
